# Prolonging Medical Procedures to Exploit the Peak‐End Rule: An Ethical Analysis

**DOI:** 10.1111/jep.70264

**Published:** 2025-08-28

**Authors:** Richard C. Armitage

**Affiliations:** ^1^ Academic Unit of Population and Lifespan Sciences, School of Medicine University of Nottingham, Clinical Sciences Building, Nottingham City Hospital Campus Nottingham UK

## Abstract

**Introduction:**

The Peak‐End Rule (PER) impacts how individuals remember events: experiences are primarily remembered according to the emotions associated with the experience's most intense moment (the peak) and those associated with its end (the end). The potential utility of exploiting the PER for improving patients' willingness to repeat unpleasant but medically useful procedures in the future has been demonstrated.

**Methods:**

This paper conducts an analysis of the ethical issues surrounding the prolongation of medical procedures to exploit the PER.

**Findings and Discussion:**

Prolonging medical procedures to exploit the PER appears to satisfy the bioethical principles of beneficence and justice. It is unclear whether, by fully informing patients that the PER is being exploited by prolonging their painful medical procedures, the effect of the PER will persist. While it is unreasonable to expect doctors to provide patients with a full explanation of every medical decision involved in their care, the degree of transparency should reflect the significance that patients are likely to attach to those decisions. It is likely that patients will consider the decision to expose them to prolonged pain without immediate clinical benefits a significant decision. Accordingly, by failing to fully inform patients that the PER is being exploited (for fear of diminishing or eradicating its effect), patients are unable to make fully informed decisions, meaning doctors fail to respect patient autonomy while also acting in an unethically paternalistic manner. Furthermore, exploiting the PER in this manner might also violate the principle of non‐maleficence, while appeals to the doctrine of double effect to justify this decision would likely be unsuccessful. A further analysis of the ethical issues surrounding the other ways in which the PER can be exploited in clinical practice, such as by reducing the intensity of pain at the peak of the experience with analgesia, is needed.

## 1 Introduction

The Peak‐End Rule (PER) is a heuristic of human psychology—a simple strategy, or mental shortcut, by which humans quickly form judgements, arrive at decisions, and identify solutions to complex problems based on a limited subset of the available information [1]. The PER impacts how individuals remember past events: experiences are primarily remembered and evaluated according to the emotions associated with the experience's most intense moment (the peak) and those associated with its end (the end). The effect occurs regardless of whether the experience has a positive (pleasant) or negative (unpleasant) valence. Other information, such as the total duration of the experience, its total net pleasantness or unpleasantness, and its average moment‐by‐moment valence) is not entirely lost, but is not used in the remembering and evaluation of the experience [2, 3, 4, 5].

Three foundational experimental psychology studies – pioneered by Daniel Kahneman, Barbara Fredrickson, and Donald Redelmeier – first demonstrated the PER. In the first [6], subjects were exposed to two unpleasant experiences: a hand was immersed in water at 14°C for 60 s; later, the hand was again immersed in water at 14°C for 60 s, but then kept in the water for an additional 30 s as the water temperature was gradually raised to 15°C. For most subjects, water at 15°C was painful, but distinctly less so than water at 14°C. Subjects were later given a choice of which experience to repeat. A significant majority chose to repeat the longer experience, thereby preferring a longer duration of pain, and greater total pain, over less (the minimal influence of the total duration is known as ‘duration neglect’). The authors concluded that “subjects chose the long trial simply because they liked the memory of it better than the alternative (or disliked it less).”

In the second study [7], the real‐time intensity of pain experienced by patients undergoing colonoscopy and lithotripsy was recorded. The retrospective evaluations of the total pain experienced during the procedure was subsequently recorded and related to the real‐time reports made during the experience. Patients' evaluations of total pain were strongly correlated with its peak intensity and with its intensity recorded during the final 3 min of the procedure. While there was substantial variation in the duration of the experience, lengthy procedures were not remembered as particularly unpleasant. The authors concluded that patients' memories of painful medical procedures are largely reflective of the pain's intensity at the peak and the end of the experience.

In the third study [8], patients undergoing colonoscopy were randomly allocated to have a standard procedure or a standard procedure with a short interval added to the end during which the tip of the colonoscope remained stationary in the rectum without manipulation. Pain during the procedure, and overall pain evaluated retrospectively, were measured. Patients who underwent the extended procedure experienced the final moments as less painful, and evaluated the entire procedure as less unpleasant. Rates of returning for repeat colonoscopy were significantly higher for those who underwent the prolonged procedure. The authors concluded that the memory failings observed in experimental conditions can also be found in clinical settings, and therefore might provide opportunities for improving patients' willingness to repeat unpleasant, but medically useful, procedures.

The implications of the PER have been demonstrated across various domains including business (such as pricing strategy) [9], travel [10], and education [11]. In clinical practice, the utility of exploiting the PER, particularly in the context of unpleasant or painful medical procedures, is demonstrated by the aforementioned studies of Kahneman, Fredrickson, and Redelmeier and, more recently, by other authors in patients undergoing cystoscopy [12] and Pap smear screening [13]. Exploiting the PER rule in clinical practice can be achieved in two ways – by reducing the intensity of pain at the peak of the experience (such as through the use of analgesia), and by reducing the intensity of the pain at the end of the experience (such as by prolonging the experience in a state of low pain). This paper will conduct an ethical analysis of prolonging medical procedures (such as scoping procedures and cervical screening) to exploit the PER. It will not examine other ways in which the PER can be exploited (such as by reducing the intensity of the pain at the peak of the experience with analgesia).

## 2 Ethical Issues Surrounding the Prolongation of Medical Procedures to Exploit the Per

### 2.1 Beneficence

The third study mentioned previously demonstrated that, by inducing the formation of less aversive memories of painful or otherwise unpleasant medical procedures, exploiting the PER by prolonging these procedures can render patients more likely to undergo repetitions of the procedures in the future [8]. Because these procedures are often used for diagnostic, screening, and surveillance (e.g., cancer surveillance) purposes, being subjected to them (when it is deemed medically necessary by clinical and epidemiological practitioners) has been shown to improve health outcomes in many cases, such as through the early identification, and therefore diagnosis and treatment, of harmful pathologies. In addition, substantial positive secondary effects might also occur. For example, the less aversive memory of an unpleasant medical procedure (such as a colonoscopy) might increase the likelihood of the patient undergoing a similar unpleasant procedure (such as a gastroscopy), or even an entirely different unpleasant procedure (such as cervical screening), when it is clinically indicated. The result of this would be a further improvement in health outcomes (while a plausible effect, no empirical evidence is currently available to support this). The promotion of such outcomes, therefore, satisfies the biomedical ethical principle of beneficence, which imposes upon doctors an obligation to act for the benefit of the patient, such as the active promotion of health [14].

### 2.2 Autonomy and Paternalism

Among the three foundational PER studies, only the third [8] – a randomised controlled trial conducted in a clinical setting – provides the strongest kind of evidence for the existence and utility of the PER in clinical practice. Each patient recruited in that study was blinded throughout (to minimise the risk of introducing bias to the study), including during the follow‐up period that investigated return rates for subsequent colonoscopy. As a result, the patients were not fully aware of the nature of the procedure they underwent. While such reduced transparency is an essential feature of effective research methodologies, the effect of full transparency was not investigated by the study. It is possible that a patient's knowledge that the painful medical procedure to which they are being subjected is being prolonged to induce the formation of less aversive memories (rather than, e.g., for additional diagnostic or therapeutic benefit) might undermine the PER and the beneficial effects it would otherwise induce (in a similar manner to the diminishment of the placebo effect once the subject becomes aware of the true nature of the intervention) [15].

Yet, doctors hold a duty to respect patient autonomy, in that they must act to preserve the patient's capacity for self‐determination and support them in making informed decisions about their care [15, 16]. Accordingly, insufficient transparency about the prolongation of painful medical procedures might prevent patients from making fully informed decisions to undergo such procedures, which seemingly violates the duty to respect autonomy. However, full transparency that would necessitate patients to be informed of all details of the procedure to which they are subjected is overly burdensome on doctors and unnecessary for the provision of safe and effective (e.g., doctors routinely ask patients to avoid eating and drinking before surgery, but do not explain the clinical reasoning behind this instruction). Instead, for patients to provide informed consent, the degree of appropriate transparency should be determined by the magnitude of the decision, meaning the degree to which it is likely to be considered significant by the patient (which English tort law holds the doctor should reasonably be aware of) [17]. In the context of prolonging medical procedures to exploit the PER, the prolonged exposure to discomfort or pain without the generation of immediate clinical benefit is likely to be considered significant by the patients undergoing those procedures. Accordingly, falling short of full transparency is likely to prevent the patient from making an informed decision to submit to a procedure that exploits the PER in this manner. Yet, while asking patients if they would accept such a procedure would render their decision more informed, the previously described risk that their knowledge of this modification might undermine the PER and the beneficial effects it would otherwise induce is repeated. Further research is required to ascertain the impact of such full transparency on health outcomes when the PER is exploited in clinical settings.

Furthermore, prolonging medical procedures to exploit the PER might raise charges of unacceptable medical paternalism. Doctors act paternalistically when they substitute the patient's judgement with their own such that medical decisions are made not by the patient, but rather by the doctor in what they consider to be in the patient's best interest. Such action generally infringes upon the doctor's duty to respect patient autonomy, and opposes the harm principle of John Stewart Mill (Mill claimed that “the only purpose for which power can be rightfully exercised over any member of a civilised community, against his will, is to prevent harm to others. His own good is not a sufficient warrant”) [18]. As such, by prolonging medical procedures to increase the likelihood of patients undergoing repeat procedures in the future (and thus promoting their future health outcomes) without the knowledge of those patients, doctors act in a paternalistic manner that is ethically unacceptable. Notably, prolonging medical procedures to exploit the PER would not amount to a ‘nudge’ (derived from Nudge Theory's ‘libertarian paternalism,’ and which preserves the subject's freedom to choose [19] and is potentially ethically acceptable) [20, 21] because, by failing to inform patients of their prolonged exposure to pain, patients are unaware that this prolongation could be refused.

### 2.3 Non‐Maleficence

Subjecting patients to prolonged medical procedures might be in violation of the ethical principle of non‐maleficence, which requires doctors to avoid intentionally harming patients.15 However, it is permissible for doctors to cause smaller harms that are necessary to protect patients from greater harms (such as the removal of a gangrenous leg, which renders the patient profoundly disabled yet prevents their death), particularly when they have the patient's consent to do so (it is also generally permissible for a doctor to act in such a manner in the best interests of a patient who lacks capacity). Yet, prolonging painful medical procedures (without the generation of any immediate clinical benefit) to exploit the PER would take place neither with the patient's consent (due to insufficient transparency), nor with the certainty that the intended positive effects will occur (since exploiting the PER only increases the likelihood of this outcome, but does not guarantee it). For the duty of non‐maleficence to be upheld, the magnitude of the harm must be smaller than that of the benefit (or, in the case of the patient undergoing repeat medical procedures in the future, the expected benefit). Accordingly, these magnitudes must be calculated in each incidence of PER exploitation in clinical practice. However, while the magnitude of the benefits could be calculated at the group level using longitudinal research and health economics techniques, the magnitude of the harm, which must be reported by patients, is likely impossible to calculate without each patient being informed that the painful procedure will be prolonged, which might diminish or eradicate the PER effect. While a doctor might consider the harm of a few extra minutes of a painful medical procedure to be smaller than the expected benefits it brings about (the increased likelihood of undergoing repeat medical procedures), not all patients would hold the same view. Accordingly, by not calculating the magnitudes of these harms and benefits in each case – which necessitates full transparency and a dedicated conversation with the patient – the doctor might violate their duty of non‐maleficence.

It might, however, be possible for this violation to be avoided via an appeal to the doctrine of double effects (DDE) [22, 23, 24]. DDE holds that it is ethically permissible for doctors to perform acts that will foreseeably produce both good and bad effects if the following conditions are satisfied simultaneously [25]:

1. The act, when considered independently of its harmful effects, is not in itself wrong;
2. The doctor intends the good effect and does not intend the harmful effect, either as a means or an end, although the doctor may foresee the harm;
3. The good effect cannot be achieved without causing the harmful effects; and
4. The harmful effects are not disproportionately large relative to the good effects that are sought [26].

By prolonging medical procedures to exploit the PER, condition 2 is not satisfied, as the doctor intends the bad effect (the prolongation of pain, albeit at a low magnitude) as a means to the good effect. Condition 3 is also not satisfied, because patients routinely undergo repeat medical procedures (albeit at lower rates) without the PER being exploited, other evidence‐based interventions exist to increase the rate at which patients undergo painful medical procedures [27, 28], and recent evidence suggests that exploiting the PER by reducing the peak of the experience might have greater utility than targeting the end [5]. It is unclear whether condition 4 is satisfied, because the magnitudes of the harmful and good effects must be calculated in each case, while condition 1 is probably satisfied, since the act (prolonging the procedure to increase the likelihood of repeated procedures) is probably not in itself wrong when considered independently of its harmful effects. Accordingly, since all four conditions are not satisfied simultaneously, exploiting the PER by prolonging medical procedures cannot be ethically justified by an appeal to the DDE.

### 2.4 Justice

The total amount of resource (including time, financial, pharmaceutical, equipment, and staffing resources) required to sufficiently care for early‐stage disease is, generally, substantially smaller than that used to sufficiently care for later‐stage disease, thereby making the former more cost‐effective than the latter [29, 30, 31]. Additionally, successful early diagnosis and treatment facilitates patients' return to productivity and workforce participation to a greater extent than late diagnosis and treatment which, particularly in tax‐payer funded socialised health systems, promotes the generation of additional resources for deployment into such systems. Accordingly, since prolonging medical procedures to exploit the PER can render patients more likely to undergo those procedures in the future and [8], as discussed in the Beneficence section, might increase the likelihood of patients undergoing similar or entirely different unpleasant procedures when clinically indicated, this is likely to lead to earlier and more cost‐effective diagnosis and treatment. In turn, this allows resources to be redistributed to benefit other patients in the health system. Additionally, this effect of increased likeliness to undergo clinically indicated medical procedures will have a disproportionately positive impact on the health outcomes of groups that currently have the lowest uptake of such procedures, thereby promoting health equity. Simultaneously, the additional resources required to prolong medical procedures, such as by an extra 2‐min during which no other intervention is carried out [12], are negligible, while prolonging each procedure by only a few minutes is unlikely to reduce the number of procedures that can take place (and, therefore, the number of patients who can benefit) in each session. Accordingly, prolonging procedures to exploit the PER satisfies the ethical principle of justice, which requires doctors to ensure that the benefits and costs of actions are fairly distributed between patients [15, 32].

## 3 Conclusion

Prolonging medical procedures to exploit the PER appears to satisfy the bioethical principles of beneficence and justice. It is unclear whether, by fully informing patients that the PER is being exploited by prolonging their painful medical procedures, the effect of the PER will persist (further research is required to ascertain this). While it is unreasonable to expect doctors to provide patients with a full explanation of every medical decision involved in their care, the degree of transparency should reflect the significance that patients are likely to attach to those decisions. It is likely that patients will consider the decision to expose them to prolonged periods of pain without immediate clinical benefits to be a significant decision. Accordingly, by failing to fully inform a patient that the PER is being exploited (for fear of diminishing or eradicating its effect), the patient is unable to make a fully informed decision, meaning the doctor fails to respect the patient's autonomy while also acting in an unethically paternalistic manner. Furthermore, exploiting the PER in this manner might also violate the principle of non‐maleficence, while appeals to the doctrine of double effect to justify this decision would likely be unsuccessful. A further analysis of the ethical issues surrounding the other ways in which the PER can be exploited in clinical practice, such as by reducing the intensity of pain at the peak of the experience with analgesia, is needed. This would be useful in the context of medical procedures that cannot be easily prolonged, such as bone marrow aspiration, while a recent meta‐analysis suggested that the effect of the peak is greater than that of the end (thereby implying that greater utility might be generated by exploiting the peak than the end) [5].

## Conflicts of Interest

R.A. declares no competing interests.

## Data Availability

Data sharing not applicable to this article as no datasets were generated or analysed during the current study.
